# Supplementary material for: In Vitro Screening of Non-Antibiotic Components to Mitigate Intestinal Lesions Caused by Brachyspira hyodysenteriae, Lawsonia intracellularis and Salmonella enterica Serovar Typhimurium
Source: Animals (Basel). 2022 Sep 9;12(18):2356. doi: 10.3390/ani12182356 (PMC9494979; doi:10.3390/ani12182356)
Supplement: Supplementary file 1 [file animals-12-02356-s001.zip › animals-1888364-Figure S1-S3.pdf]

Supplementary material 2

1. Exposure to Pathogens Induced Microscopic Changes in Colon Explants

For evaluation of the screening method used in this study, the findings below represent the comparison between all explants from the pathogen control group (PCG) and compound control group (CCG) groups for a given pathogen.

1.1. Brachyspira Hyodysenteriae

No significant difference was observed for the percentage of healthy epithelium between PCG and CCG (Figure A1A). In contrast, a significantly thicker mucus layer was found in the PCG (early time-point and late time-point, Figure A1B) and significant higher levels of tumor necrosis factor-α (TNF-α), interleukin-1α (IL-1α) and interferon-γ (INF-γ) mRNA expression were found at the late time-point (Figure S1A).

1.2. Lawsonia Intracellularis

No differences between groups in epithelial coverage or gene expression were observed at either time-point (Figures S1C and S2B).

1.3. Salmonella Enterica Serovar Typhimurium

CCG explants had significant more healthy epithelial coverage than the PCG samples at both time-points (Figure A1D). Late time-point PCG samples had increased IL-1α mRNA expression (Figure S2C).

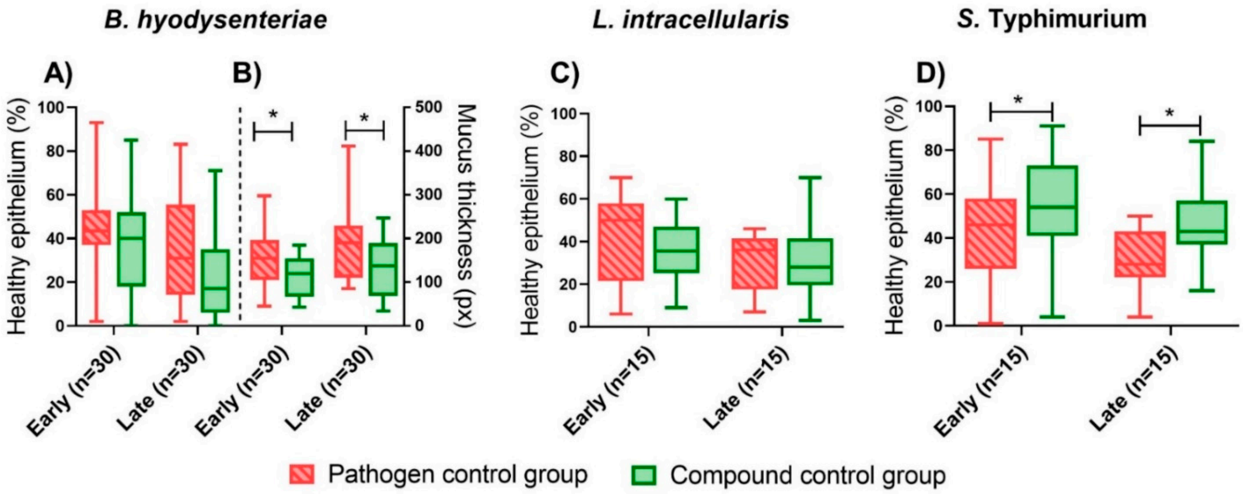

Figure S1. Microscopic explant health comparison between the compound control group (CCG), including all compound groups combined, and pathogen control group (PCG) for evaluation of screening method used in current study. Percentage of healthy epithelium (A) and mucus layer thickness (B) in explants harvested from pigs and exposed to *B. hyodysenteriae* (PCG) or controls (CCG). C) Percentage of healthy epithelium on explants from pigs exposed to *L. intracelullaris* (PCG) or controls (CCG). D) Percentage of healthy epithelium on explants from pigs exposed to *S.* Typhimurium (PCG) or controls (CCG). Boxplots depict the median ± standard deviation of the median. Whiskers depict the minimum and maximum values. Stars denote significant difference between groups (P ≤ 0.05).

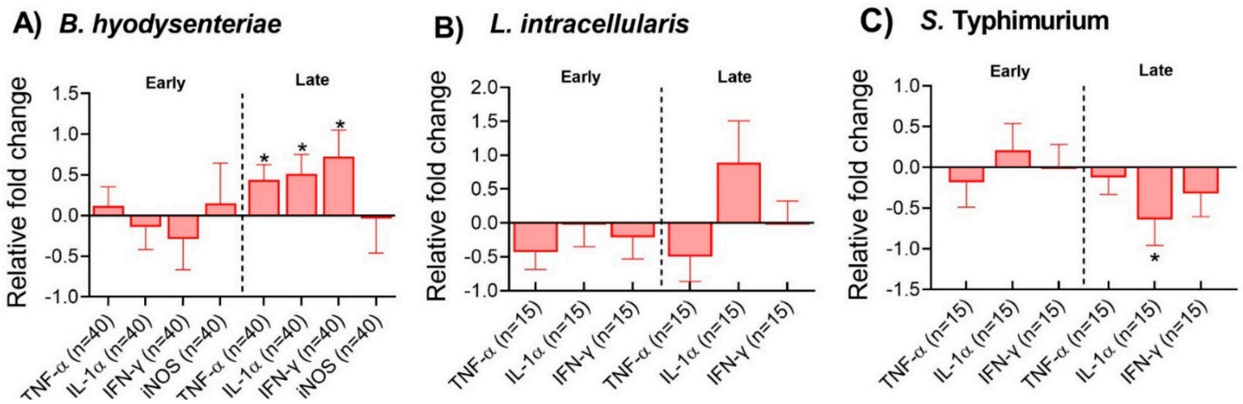

Figure S2. Change in mRNA levels between the compound control group (CCG), including all compound groups combined, and pathogen control group (PCG) for evaluation of screening method used in current study. CCG was used as the reference group. A) Gene expression levels for explants from pigs exposed to *B. hyodysenteriae*. B) Gene expression levels for explants from pigs exposed to *L. intracellularis*. C) Gene expression levels for explants from pigs exposed to *S.* Typhimurium. Each bar represents mean fold-change, whiskers represent the standard deviation from the mean. Star denotes significant difference (P ≤ 0.05).

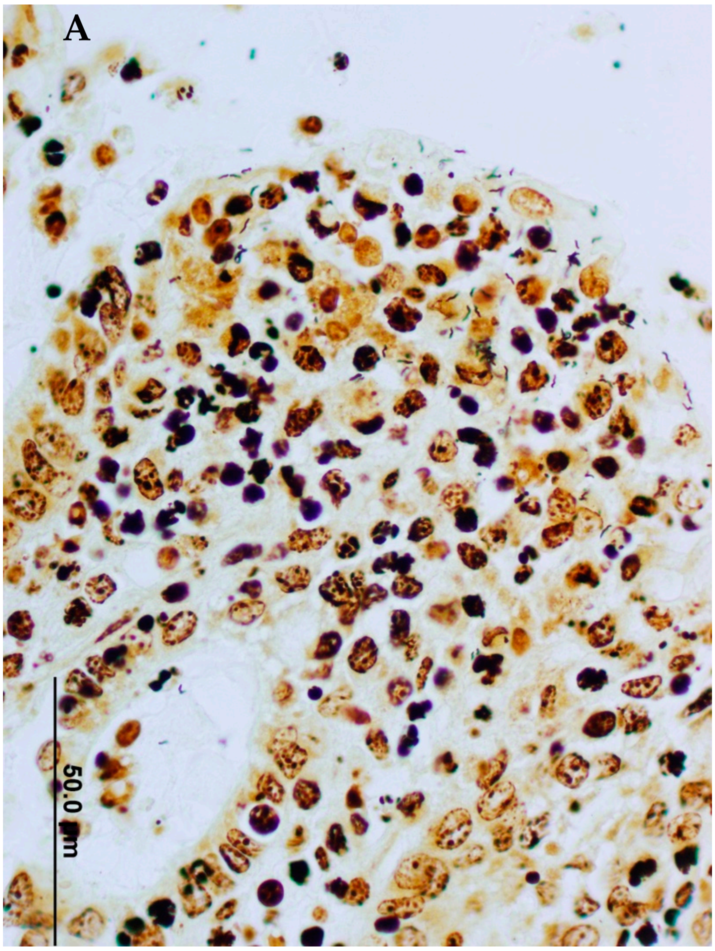

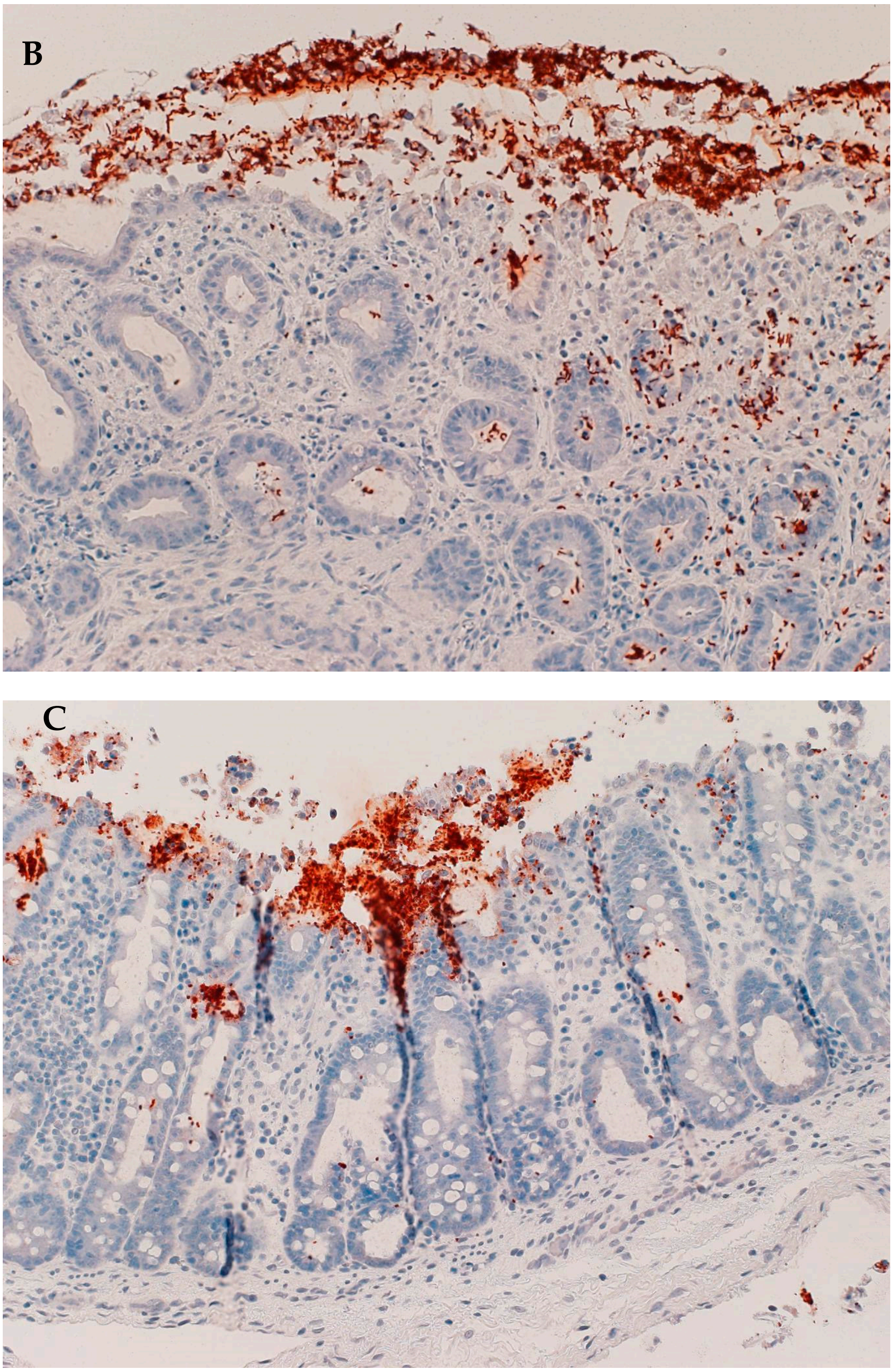

Figure S3. Examples of Warthin-faulkner-stained explant inoculated with *L. intracellularis* (A), immunohistochemistry for *B. hyodysenteriae* (B) and *S.* Typhimurium (C)
