# Supplementary material for: In Vitro Screening of Non-Antibiotic Components to Mitigate Intestinal Lesions Caused by Brachyspira hyodysenteriae, Lawsonia intracellularis and Salmonella enterica Serovar Typhimurium
Source: Animals (Basel). 2022 Sep 9;12(18):2356. doi: 10.3390/ani12182356 (PMC9494979; doi:10.3390/ani12182356)
Supplement: Supplementary file 1 [file animals-12-02356-s001.zip › animals-1888364-Table S1-S2.pdf]

Supplementary material 1

Additional information and description of the total number of explants used per animal per analysis

Table S1 Description of the total number of explants used per animal for each compound (F, L, P, and S) and pathogen (*B. hyodysenteriae*, *L. intracellularis* and *S.* Typhimurium).

**Number of animals**	**Combination groups**	**RT-PCR**		**Histopathology**		**Total of explants per combination groups**
		Early	Late	Early	Late	
5	Pathogen control group (PCG)	1	1	1	1	4
	Compound control group (CCG)	1	1	1	1	4
	Treatment group (TG)	1	1	1	1	4

Table S2 Description of the total number of explants used per animal for compound D only challenged with *B. hyodysenteriae*.

**Number of animals**	**Combination groups**	**RT-PCR**		**Histopathology**		**Total of explants per combination groups**
		Early	Late	Early	Late	
5	Pathogen control group (PCG)	4	4	2	2	12
	Compound control group (CCG)	4	4	2	2	12
	Treatment group (TG)	4	4	2	2	12
